# The association between viewing cigarette health warning labels and intention to quit smoking among Chinese adult smokers: support for including health outcome content and culturally specific messages

**DOI:** 10.1186/s12889-023-15718-4

**Published:** 2023-05-11

**Authors:** Qinghua Nian, Jeffrey J Hardesty, Joanna E Cohen, Ryan D Kennedy

**Affiliations:** Institute for Global Tobacco Control, Department of Health, Behavior & Society, Johns Hopkins Bloomberg School of Public Health, Johns Hopkins University, 2213 McElderry St, 4th Floor, Baltimore, MD 21205 USA

**Keywords:** Health warning labels, Health communication, Intention, Quit smoking, Chinese smokers, Cigarette, Policy, Low- and middle-income country

## Abstract

**Background:**

Tailored themes of pictorial health warning labels (HWLs) that considers specific cultural dimensions and within a specific policy/historical context can motivate behavior change, such as provoking smokers to think about quitting. Currently in China, the HWLs on cigarettes are text-only. This study designed pictorial HWLs using different themes to explore and examine the association between viewing pictorial HWLs and participants’ intention to quit smoking. The themes included: (1) Self-harm from using cigarettes, (2) Harming family or children with secondhand smoke, (3) Complying with existing smoke-free policies, and (4) Cigarette gift giving practices.

**Methods:**

A cross-sectional randomized experimental survey was conducted among 1,625 smokers in Beijing (n = 545), Shanghai (n = 541), and Shenzhen (n = 539) during 2017. Before and after viewing eight pictorial HWLs of one theme, participants were asked if they had plans to quit smoking within the next month, within the next 6 months, beyond the next 6 months, or not at all. Ordinal logistic regression, Wilcoxon and Chi-square tests were used to analyze the data.

**Results:**

After viewing the HWLs, 434 participants (26.9%) reported an intention to quit smoking sooner, 987 (61.2%) reported the same intention to quit, and 191 (11.8%) reported an intention to quit later. The pre-post change in intention to quit was statistically significant among all participants and participants under each theme (p > 0.05). Participants who rated the effectiveness of the HWL communicating how secondhand smoke harms children had 1.13 (95% CI 1.01–1.27) greater odds of reporting an intention to quit sooner compared to those viewing labels from the other themes, adjusting for covariates. Female participants and participants with annual household income more than 100,000RMB had 1.39 (95% CI 1.14–1.69) and 1.29 (95% CI 1.04–1.60) greater odds of reporting an intention to quit sooner compared to their counterparts across the entire sample.

**Conclusions:**

Findings of this study provide evidence of an association between all four pictorial HWL themes and smokers’ intention to quit smoking. These findings can help inform what HWL themes might be appropriate when China implements pictorial health warning labels.

## Background

Smoking is the leading cause of preventable death in China [1, 2] which causes over 1 million deaths [3, 4]. There are approximately 307.6 million Chinese cigarette smokers (including 52.4% of men and 2.3% of women) [5]. The Healthy China 2030 strategy establishes targets for tobacco control, including a reduction in smoking prevalence from 27.7% to 2015 to 20% by 2030 and an increase in public awareness of smoking-related health risks [6]. Key public health measures must be implemented to achieve this target.

Pictorial health warning labels (HWLs) on cigarette packages are a highly cost-effective tobacco control measure [7, 8]. HWLs can communicate information about the health risks of cigarettes and cause smokers to think more about the health harms of using cigarettes [9–13]. HWLs with detailed risk information and pictures on cigarette packages can increase level of fear and disgust for the health hazards of smoking and provoke thoughts and motivations to quit smoking [12, 14]. Studies in many countries including Thailand, Malaysia, Mauritius, Australia and Canada suggest that pictorial HWLs are effective in promoting interest in quitting smoking [15–17]. Levy et al. estimated that pictorial HWLs may reduce smoking prevalence by 5% in US over the short term and by 10% over the long term [18]. ITC Project investigators compared the smoking rates in Canada before and after the implementation of pictorial HWLs, taking into account the price of cigarettes, and found that pictorial HWLs were effective in decreasing overall smoking rates by 2.9–4.7% [19].

China enacted its first HWL-related legislation in 1991 which required a text health warning on one of the cigarette pack side panels stating “smoking is harmful to your health” in Chinese [20]. In 2005, China ratified the World Health Organization Framework Convention on Tobacco Control (WHO FCTC) which requires large and clear HWLs to cover at least 30% of the package. As a party to WHO FCTC, new regulations were implemented in 2008 which increased the warning label coverage to 30%, placed the health warning on the front in Chinese and back in English, and added one more sentence – “quit[ting] smoking early is good for your health” following “smoking may harm your health” in the message. In 2012, several changes were made to Chinese HWLs again increasing the minimum text font size from 2.0 to 4.0 mm and changing the English text on the back of the pack to Chinese characters [21]. Both iterations of the latest Chinese HWLs are without information on specific smoking-related diseases and provide no useful information on how to access supports for cessation [22]. The 2018 GATS China report suggests that two-thirds of smokers who had seen a HWL did not consider quitting [5]. Elton-Marshall et al. (2015) found that most adult smokers from six cities in China thought that the text-only HWLs did not provide adequate health information about the harms of smoking [23]. Fong et al. (2010) conducted an experimental study of smokers and non-smokers in four Chinese cities to explore the effects of HWLs on quitting smoking and smoking prevention [24]. The study suggested that the text-only Chinese HWLs were not effective in promoting interest in quitting smoking and convincing Chinese adolescents not to initiate smoking. One cross-sectional survey study in Hangzhou suggested that more than 50% of Chinese smokers reported that they intended to quit smoking after viewing graphic images depicting individuals with lung diseases or mouth cancer caused by smoking, or a child or a woman suffering from secondhand smoke [25]. Wang et al. (2021) conducted a study in Songjiang district and Fengxian district of Shanghai and over half current smokers in the study reported an intention to quit smoking as a result of seeing a pictorial HWL on cigarette packs that depicted oral and throat cancer caused by smoking [26]. These researchers also found female smokers had higher smoking cessation intention compared to male smokers due to the pictorial HWLs.

Cultural differences exist in relation to the meaning of visual images in anti-cigarette use campaigns in the US [27, 28]. This suggests that the themes of pictorial HWLs (i.e., the combination of text and images) need to be designed with considerations for specific cultural dimensions and within a specific policy/historical context. Tailored public health messages can motivate behavior change. [29, 30]. It is important to note that most cigarette HWL studies, have been conducted in high income [31–33]. In designing pictorial HWLs for China, content that includes specific health effects from smoking presented in ways that consider relevant policy and culture can be credible and effective [34].

The current study designed HWLs considering common practices for pictorial HWL design in other jurisdictions, including information about health consequences to smokers (such as cancer and lung diseases) and to non-smokers from exposure to secondhand smoke (SHS). People who live in China are often described as being more collectivist, compared to people who live in western countries [35]. Among Chinese smokers, concern about the health of family members is one of the most commonly reported reasons for quitting [36, 37]. HWLs could be effective if communicating the harms of SHS on family or children since this aligns with the strong sense of obligation to family held in Chinese culture [36, 38]. We also consider the practices around smoking and smoke-free policy in China. Efforts to pass subnational smoke-free legislation has made progress in China, although draft smoke-free legislation at the national level has been placed on hold since 2016 due to resistance from the State Tobacco Monopoly Administration. Large cities in China, such as Beijing in 2015, Shanghai in 2017 and Shenzhen in 2017, have adopted 100% smoke-free policies as per standards outlined by WHO FCTC Article 8 but there have been challenges with their adoption and enforcement [39–43]. HWLs about complying with smoke-free policies could remind smokers about the dangers of SHS and the responsibility for clean air. In addition, China-specific cultural practices around tobacco products were considered. It is a tradition in China that people give cigarettes as a gift to others to establish or maintain relationships during holidays and special occasions [44]. An online nationwide survey between September 2017 and August 2018 showed that approximately 90% of smokers and 60% of non-smokers reported experiences of gifting cigarettes [45]. HWLs linking cigarette gifting to lung diseases could highlight the health harms of this practice.

China has the largest population of people who smoke in the world and smoking cessation is an important behavior to support personal and public health. Previous studies conducted in China used existing graphic images depicting health harms from jurisdictions outside China. Research is needed to explore the effectiveness of pictorial HWL themes designed specifically for China and their impact on Chinese smokers’ interests in quitting smoking. This study sought to examine a potential association between viewing pictorial HWL themes that account for Chinese culture, values, and public health goals and Chinese smokers’ intention to quit smoking and observe whether the association varied by smokers’ sociodemographic characteristics.

## Methods

### Sample and recruitment

This cross-sectional study used a Chinese research firm to collect survey data from adults (aged 18 and older) living in Beijing, Shanghai, and Shenzhen during 2017. All three cities have ordinances banning smoking in indoor workplaces, public places, and transit environments. Recruiters, screeners and data collectors from the research firm participated in an in-person training in each of the survey cities; the trainings were provided by a co-investigator and two graduate students from the Johns Hopkins Bloomberg School of Public Health. Participants were recruited in public retail venues, such as malls, and were screened for their eligibility. They were eligible to participate if they met the following criteria: (1) Residency (lived in survey city for at least one year), (2) Not professionally affiliated with the tobacco industry, and (3) Smokers. Male smokers were defined as smoking daily, whereas female smokers were defined as smoking any day in the last 30 days. The 2018 GATS data showed that 50.5% of men and 2.1% of women were current smokers; and 44.4% of men and 1.6% of women were current daily smokers [5]. With consideration of the big difference between men and women, we used a relatively less strict definition for female smokers in order to recruit a sample of women most likely to regularly encounter cigarette HWLs in China. Both male and female smokers reported smoking more than 100 cigarettes in their lives. Quotas were used for age (50% 18–39 years old, 50% 40 and older) and sex (50% male, 50% female) to ensure that each analysis group had a minimum of 400 participants to achieve statistical power at 0.8 (α = 0.05) for an effect size of 0.2. Participants received a voucher for 50 RMB (~$7.37 USD) for their time.

### Health warning label theme design

**Fig. 1:**
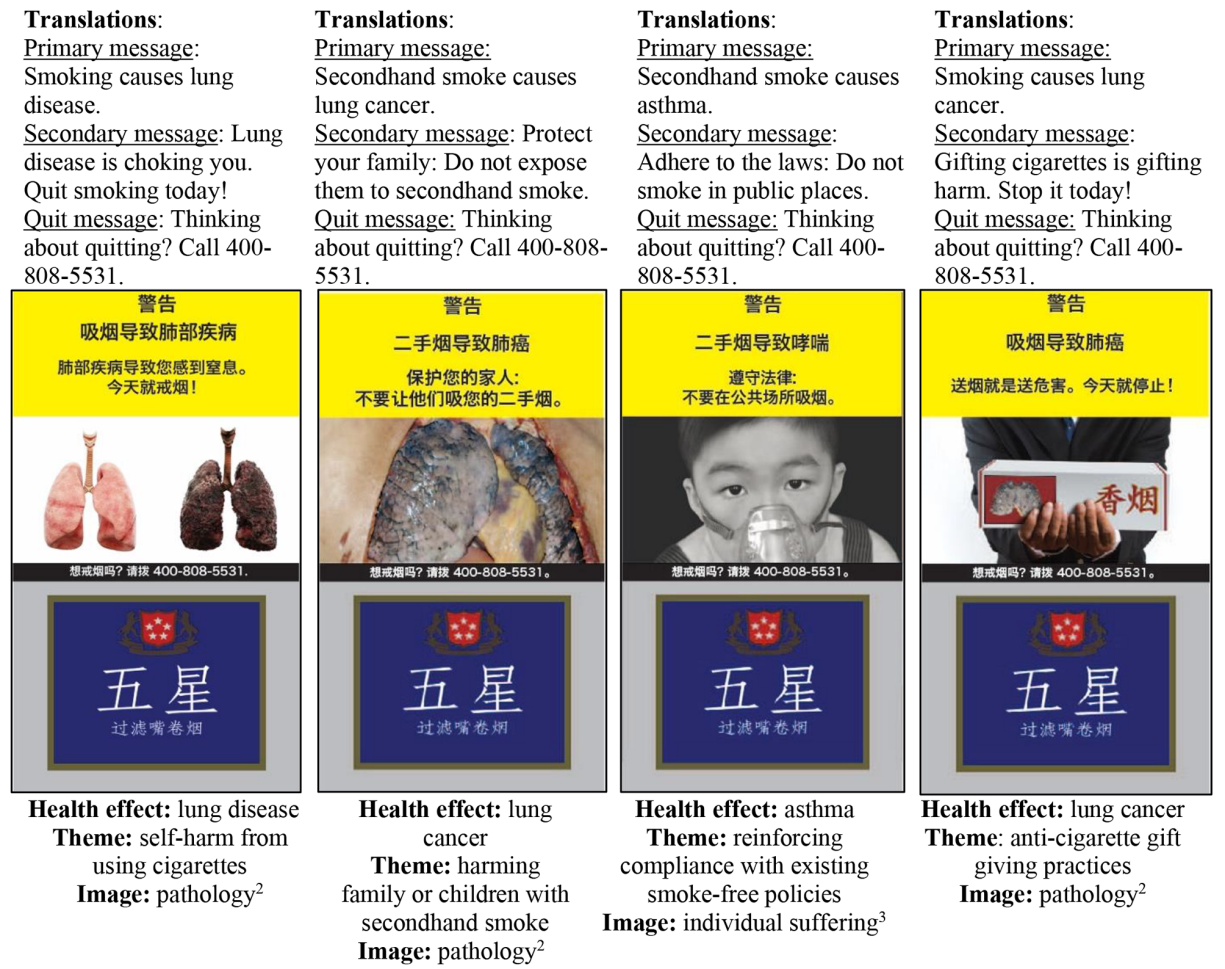
Sample of pictorial health warning labels (HWLs) ^1^ Each HWL in the figure represents one of the eight HWLs from each theme. Supplemental figures include all 32 HWLs viewed ^2^ Courtesy of Dr. Prakit, Action on Smoking and Health Foundation, Thailand ^3^ ©Her Majesty the Queen in Right of Canada, represented by the Minister of Health (2013)

Four themes, including: (1) Self-harm from using cigarettes, (2) Harming family or children with SHS, (3) Reinforcing compliance with existing smoke-free policies, and (4) Anti-cigarette gift giving practices were designed. Within each theme, there are eight HWLs including warning label images and a primary message, i.e., “Warning: Smoking causes lung cancer” or “China CDC: Smoking causes lung cancer”, secondary message, i.e., “Let your lungs heal: Quitting today may improve your breathing within a few weeks”, and a quit message, i.e., “Thinking about quitting? Call 400-808-5531” (Fig. 1). The health effects were limited to diseases caused by both smoking and secondhand smoke (lung cancer and asthma). The questionnaire and cigarette pack visual stimuli were designed in English, then translated into Chinese. We also pre-tested these with multiple native speakers to ensure translations were accurate, and the training provided to data collectors and data collection were conducted in Chinese.

**Fig. 2:**
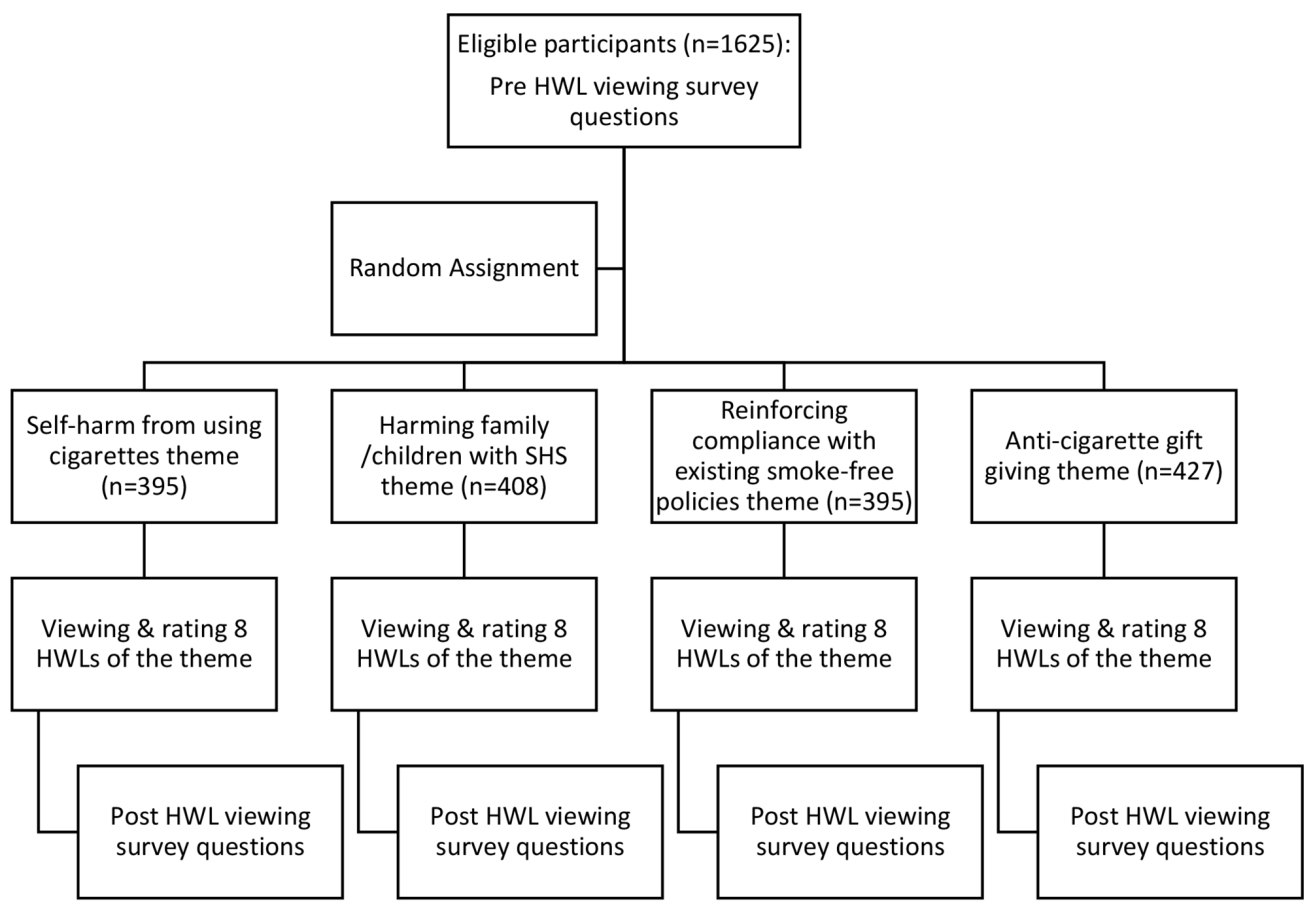
Procedures to collect data from eligible participants

Participants were randomly assigned to one theme of HWL (Fig. 2). Then participants viewed eight HWLs corresponding to the theme, assessed the effectiveness of themed HWLs, and responded to the same intention to quit questions before and after viewing the HWLs. The study staff showed participants the laminated photos containing individual HWLs for the corresponding questions. The participant looked at the image (no instructions), read the question, answered the question, and moved on to the next. The exposure was not for a set period of time because we wanted this to more closely mimic the real-world experience of seeing these HWLs on the pack.

### Measures

Intention to quit smoking was assessed by asking “Are you planning to quit in the next month, next 6 months, beyond 6 months, or not at all?”. A 3-category variable – “intention to quit sooner”, “no change in intention to quit”, and “intention to quit later” was created to measure the change in participants’ responses before and after viewing eight pictorial HWLs. Participants were classified as having an “intention to quit sooner” if they reported their intention to quit changing from “I do NOT plan to quit smoking” to “Within the next month”, “Within the next 6 months”, or “Beyond 6 months”; or reported that they intended to quit earlier post-exposure to pictorial HWLs (e.g., responses changed from planning to quit “Beyond 6 months” to “Within the next month”). Participants were classified as “no change of intention to quit” if they reported their intention the same pre- and post-exposure to pictorial HWLs. Otherwise, they were classified as having an “intention to quit later”.

Effectiveness of themed HWLs was measured on a scale of 1 to 10 (1=“not at all”, 10=“extremely”) for whether the themed label (1) was credible, (2) might prevent the gifting of cigarettes, (3) made people concerned about smoking near their family, (4) made people concerned about smoking near children, (5) made people more likely to comply with public smoking bans, and (6) made people think about quitting.

Four sociodemographic characteristics were dichotomized for the analysis: gender (male, female); age (> 40, >=40 years old); annual household income ( > = 100,000 RMB, > 100,000 RMB); and educational attainment (> college degree, college degree).

### Statistical analysis

Statistical analyses were performed using SPSS® for Windows®, version 27.0 (SPSS Inc., Chicago, IL, USA). Descriptive statistics, including means and standard deviations or frequency distributions and 95% confidence intervals (CI), were used to describe the studied population and variables. Wilcoxon tests were performed to detect statistically significant differences in participants’ intention to quit smoking pre- and post-exposure to pictorial HWLs. The differences among participants by sociodemographic characteristics or HWL themes were examined by Chi-square (Chi-sq) tests and Kruskal-Wallis in relation to intention to quit smoking. Further, multiple linear regression was used to investigate the association between intention to quit smoking after exposure to the designed HWL themes and effectiveness of HWLs controlling for participants’ pre-exposure intention to quit smoking. Sociodemographic variables (i.e., age, gender, annual household income and education level) and the themes of HWL were included in the regression model as covariates. For each analysis, a 2-sided p > 0.05 was used to determine statistical significance.

## Results

### Sample

In total, there were 1,625 smoker participants who lived in Beijing (n = 545), Shanghai (n = 541) and Shenzhen (n = 539) (Table 1). Most of the participants (83.7%) had completed high school or had a higher educational level. Approximately half of participants (48.5%) had an annual household income under or equal to 100,000 RMB (~$15,500 USD). There were no significant differences in sociodemographic characteristics of participants by the HWL theme assigned.

**Table 1:** Sociodemographic characteristics of survey participants (smokers) by pictorial HWL theme

	Total (N = 1625)	Self-harm from using cigarettes (n = 395)	Harming family /children with SHS (n = 408)	Reinforcing compliance with existing smoke-free policies (n = 395)	Anti-cigarette gift giving	P value (n = 427)
	n (%)	n (%)	n (%)	n (%)	n (%)	
**City**						0.95
Beijing	545 (33.5)	138 (34.9)	137 (33.6)	132 (33.4)	138 (32.3)	
Shanghai	541 (33.3)	123 (31.1)	140 (34.3)	130 (32.9)	148 (34.7)	
Shenzhen	539 (33.2)	134 (33.9)	131 (32.1)	133 (33.7)	141 (33.0)	
**Gender**						0.87
Male	814 (50.1)	202 (51.1)	199 (48.8)	195 (49.4)	218 (51.1)	
Female	811 (49.9)	193 (48.9)	209 (51.2)	200 (50.6)	209 (48.9)	
**Age**						0.51
18–29	381 (23.4)	95 (24.1)	97 (23.8)	97 (24.6)	92 (21.5)	
30–39	434 (26.7)	111 (28.1)	117 (28.7)	92 (23.3)	114 (26.7)	
40–49	469 (28.9)	120 (30.4)	101 (24.8)	124 (31.4)	124 (29.0)	
50–59	267 (16.4)	56 (14.2)	71 (17.4)	64 (16.2)	76 (17.8)	
60+	74 (4.6)	13 (3.3)	22 (5.4)	18 (4.6)	21 (4.9)	
**Annual household income**						0.70
< 50,000 RMB	207 (12.7)	44 (11.1)	59 (14.5)	50 (12.7)	54 (12.6)	
50,000-100,000 RMB	317 (19.5)	81 (20.5)	65 (15.9)	81 (20.5)	90 (21.1)	
100,000 RMB	264 (16.2)	58 (14.7)	72 (17.6)	63 (15.9)	71 (16.6)	
100,000-150,000 RMB	366 (22.5)	83 (21.0)	97 (23.8)	94 (23.8)	92 (21.5)	
150,000-200,000 RMB	284 (17.5)	79 (20.0)	68 (16.7)	68 (17.2)	69 (16.2)	
> 200,000 RMB	185 (11.4)	50 (12.7)	47 (11.5)	37 (9.4)	51 (11.9)	
Missing	2 (0.1)	0 (0)	0 (0)	2 (0.5)	0 (0)	
**Education**						0.37
Primary school or less	48 (3.0)	10 (2.5)	13 (3.2)	12 (3.0)	13 (3.0)	
Secondary school	212 (13.0)	57 (14.4)	46 (11.3)	43 (10.9)	66 (15.5)	
High school or voc school	601 (37.0)	131 (33.2)	160 (39.2)	147 (37.2)	163 (38.2)	
College or more	759 (46.7)	196 (49.6)	189 (46.3)	191 (48.4)	183 (42.9)	
Missing	5 (0.3)	1 (0.3)	0 (0)	2 (0.5)	2 (0.5)	

### Intention to quit smoking before and after viewing pictorial HWL themes

Before viewing the pictorial HWL themes, nearly half of the participants (48.1%) reported that they did not plan to quit smoking, 23.7% intended to quit beyond six months, 19.5% intended to quit within the next six months, and 8.4% intended to quit within the next month (Table 2). After viewing the pictorial HWL themes, less than one-third (32.6%) of participants reported they did not plan to quit smoking, which was about 15% points less than the pre-exposure percentage. The post-exposure percentage of participants intending to quit within the next six months and beyond six months increased from 23.7 to 35.2% and from 19.5 to 23.4%, respectively; and the post-exposure percentage of those intending to quit within the next month stayed about the same. Similar movements were detected in each subgroup and by HWL theme. The pre-post exposure changes among all participants and subgroups by HWL theme were statistically significant. The HWL theme viewed did not have a significant influence on the pre-post change.

**Table 2:** Participants’ intention to quit smoking before and after viewing pictorial HWLs

Are you planning to quit smoking?	Total		Self-harm from using cigarettes		Harming family/children with SHS		Reinforcing compliance with existing smoke-free policies		Anti-cigarette gift giving	
	(N = 1625)		(n = 395)		(n = 408)		(n = 395)		(n = 427)	
	Pre	Post	Pre	Post	Pre	Post	Pre	Post	Pre	Post
	n (%)	n (%)	n (%)	n (%)	n (%)	n (%)	n (%)	n (%)	n (%)	n (%)
I do NOT plan to quit smoking	782 (48.1)	530 (32.6)	193 (48.9)	118 (29.9)	183 (44.9)	123 (30.1)	198 (50.1)	143 (36.2)	208 (48.7)	146 (34.2)
Beyond 6 months	385 (23.7)	572 (35.2)	85 (21.5)	138 (34.9)	96 (23.5)	154 (37.7)	104 (26.3)	138 (34.9)	100 (23.4)	142 (33.3)
Within 6 months	317 (19.5)	381 (23.4)	86 (21.8)	99 (25.1)	85 (20.8)	96 (23.5)	65 (16.5)	85 (21.5)	81 (19.0)	101 (23.7)
Within the next month	137 (8.4)	132 (8.1)	30 (7.6)	36 (9.1)	42 (10.3)	33 (8.1)	27 (6.8)	27 (6.8)	38 (8.9)	36 (8.4)
Missing	4 (0.2)	10 (0.6)	1 (0.3)	4 (1.0)	2 (0.5)	2 (0.5)	1 (0.3)	2 (0.5)	0 (0)	2 (0.5)
Wilcoxon signed-rank test	z=-9.22 p > 0.001		z=-5.83 p > 0.001		z=-3.32 p > 0.01		z=-4.57 p > 0.001		z=-4.64 p > 0.001	

We also examined whether participants reported an intention to quit sooner, no change of intention to quit, or an intention to quit later after viewing the pictorial HWL themes based on their pre-post responses of intention to quit smoking by city, sociodemographic characteristics, HWL theme and perceived effectiveness of HWL themes (Table 3). Overall, viewing the pictorial HWLs had a positive correlation with participants’ intention to quit smoking. Approximately 27% of participants (26.9%) reported an intention to quit sooner; 61.2% reported no change of intention to quit; and 11.8% reported an intention to quit later. The subgroup analyses suggested that pre-post change of quitting intention varied by city, gender, annual household income and perceived effectiveness of HWL themes. A significantly lower percentage of participants in Beijing, females, or participants with more annual household income, reported an intention to quit later compared to their counterparts. Participants who reported an intention to quit sooner rated the effectiveness of HWL themes (including credibility, stopping people from gifting cigarettes, making people think about quitting, making people concerned about smoking near family and children and make people more likely to comply with public smoking bans) the highest compared to participants who reported no change or an intention to quit later (p > 0.05). Among participants who viewed the self-harm themed pictorial HWLs, 30.3% reported an intention to quit sooner. This was the highest performing theme as compared to participants who viewed harming family or children with secondhand smoke theme (25.4%), reinforcing compliance with existing smoke-free policies theme (27.0%) and anti-cigarette gift giving practices theme (25.2%), although the differences were not statistically significant (p = 0.74).

**Table 3:** Characteristics and perceptions of participants by changes in participants’ intention to quit smoking before and after viewing pictorial HWLs

	Intention to quit later	No change	Intention to quit sooner	P value
	n (%)	n (%)	n (%)	
Total	191 (11.8)	987 (61.2)	434 (26.9)	
**City**				> 0.01
Beijing	41 (7.6)	345 (64.0)	153 (28.4)	
Shanghai	58 (10.7)	337 (61.9)	149 (27.4)	
Shenzhen	92 (17.4)	305 (57.7)	132 (25.0)	
**Gender**				> 0.01
Male	102 (12.7)	519 (64.5)	184 (22.9)	
Female	89 (11.0)	468 (58.0)	250 (31.0)	
**Age**				0.66
> 40 years old	95 (11.8)	486 (60.3)	225 (27.9)	
>=40 years old	96 (11.9)	501 (62.2)	209 (25.9)	
**Annual household income**				> 0.01
>=100,000 RMB	119 (15.2)	461 (59.0)	201 (25.7)	
> 100,000 RMB	71 (8.6)	525 (63.3)	233 (28.1)	
**Education**				0.21
High school or less	112 (13.1)	522 (61.1)	221 (25.8)	
College or more	79 (10.5)	461 (61.3)	212 (28.2)	
**HWL Themes**				0.74
Self-harm	43 (11.0)	229 (58.7)	118 (30.3)	
Harming family/children	51 (12.6)	251 (62.0)	103 (25.4)	
Smoke-free	47 (12.0)	239 (61.0)	106 (27.0)	
Anti-cigarette gift giving	50 (11.8)	268 (63.1)	107 (25.2)	
**Effectiveness of HWL theme**	**Mean (SD)**	**Mean (SD)**	**Mean (SD)**	**P value**
The warning label is credible.	7.07 (1.63)	7.04 (1.86)	7.36 (1.59)	0.01
The warning label would stop me from gifting cigarettes.	7.10 (1.69)	6.68 (2.13)	7.14 (1.81)	> 0.01
The warning label makes me think about quitting.	7.05 (1.69)	6.55 (2.16)	7.15 (1.67)	> 0.01
The warning label makes me concerned about smoking near my family.	7.24 (1.64)	7.05 (1.93)	7.49 (1.63)	> 0.01
The warning label makes me concerned about smoking near children.	7.36 (1.70)	7.36 (1.91)	7.79 (1.62)	> 0.01
The warning label makes me more likely to comply with public smoking bans.	7.24 (1.59)	7.17 (2.01)	7.54 (1.70)	> 0.01

### Association between change in quit intention and effectiveness of pictorial HWL theme

As shown in Table 4, intention to quit earlier after exposure to a HWL theme was significantly associated with the effectiveness of the HWL theme regarding making people think about quitting and concerned about smoking near children and significantly negatively associated with effectiveness of the HWL theme regarding stopping people from gifting cigarettes, controlling for participants’ pre-exposure intention to quit smoking and other covariates (p > 0.05). Female participants were more likely to show an intention to quit sooner after viewing HWL theme compared to their counterparts. The effectiveness of HWL theme regarding making people concerned about smoking near family was not included in the model due to a high collinearity with other independent variables (VIF = 13.3).

**Table 4:** Predictors of intention to quit smoking after exposure to the designed HWL theme

	Regression coefficient (95% CI)	P value
Pre-exposure intention to quit smoking	0.52 (0.48–0.55)	> 0.001
Gender		
Male	1.00 (Ref)	
Female	**0.15 (0.08–0.21)**	**> 0.001**
**Age**		
> 40 years old	1.00 (Ref)	
>=40 years old	-0.04 (-0.11-0.03)	0.268
**Annual household income**		
>=100,000 RMB	1.00 (Ref)	
> 100,000 RMB	-0.06 (-0.13-0.01)	0.101
**Education**		
High school or less	1.00 (Ref)	
College or more	0.00 (-0.08-0.07)	0.929
**HWL theme**		
Anti-cigarette gift giving	1.00 (Ref)	
Self-harm from using cigarettes	0.05 (-0.04-0.15)	0.252
Harming family /children with SHS	-0.05 (-0.14-0.05)	0.320
Reinforcing compliance with existing smoke-free policies	-0.05 (-0.14-0.05)	0.345
**Effectiveness of HWL theme**		
The warning label is credible.	-0.04 (-0.09-0.01)	0.087
The warning label would stop me from gifting cigarettes.	**-0.04 (-0.07-0.00)**	**0.030**
The warning label makes me think about quitting.	**0.15 (0.11–0.19)**	**> 0.001**
The warning label makes me concerned about smoking near children.	**0.05 (0.01–0.09)**	**0.015**
The warning label makes me more likely to comply with public smoking bans.	0.00 (-0.04-0.04)	0.853
**R-squared**	0.49	

## Discussion

To the best of our knowledge, this is the first study in China that examined changes in smokers’ intention to quit smoking in response to HWL themes designed specifically for the Chinese context. The findings of this study suggest an association between viewing pictorial HWLs and an intention to quit smoking. Also, the effectiveness of the HWLs that communicated concern about smoking near children is positively associated with smokers’ quitting interests. Overall, 26.9% smokers reported an earlier intention to quit after viewing the pictorial HWL theme. Among smokers who initially did not plan to quit smoking (48.1%), almost one half reported that they had an intention to quit after viewing the pictorial HWLs across all themes.

Pictorial HWLs can reach large numbers of smokers and non-smokers and have the potential to decrease communication inequality across various sociodemographic subgroups [46–48]. This study suggests that the association between viewing pictorial HWLs and intention to quit smoking may not be impacted by age and education level. Given smoking is higher among those with lower educational attainment [49], there is a need for public health measures in China that are equally effective across all educational attainment levels. Pictorial HWLs are considered an effective measure to mitigate tobacco-related inequalities related to education [50]. Cantrell et al. (2013) found a greater impact of pictorial HWLs compared to the text-only warning on credibility and intention to quit smoking; the impact was consistent across diverse socioeconomic populations [48].

This study also found that female smokers and smokers with an annual household income above 100,000RMB were more likely to report interest in quitting smoking sooner than their counterparts after viewing the pictorial HWL themes. The finding about females was in line with previous evidence that female smokers had stronger responses to pictorial HWLs than male smokers [26, 51]. Although the prevalence of smoking among women in China is low (2.4%), several studies suggest that the number of female smokers has increased recently in some regions [5, 52, 53]. The adoption and implementation of pictorial HWLs that include harms of specific diseases may improve knowledge of the harms associated with smoking and elicit smoking cessation activity among women and men. Further research is needed to understand if pictorial HWLs further discourages smoking initiation.

Assessing the impact of different pictorial HWL themes is critical to evaluating smoking cessation intentions [54]. This study designed four HWL themes for participants to view. These pictorial HWL themes addressed the obligation to family and children, need for compliance with smoke-free policies, and the negative effects of cigarette gifting in addition to individual harms of smoking in China. The results suggest that each of these HWLs themes were associated with provoking quit-related thoughts. After viewing the self-harm, harming family or children with SHS, reinforcing compliance with existing smoke-free policies, and anti-cigarette gift giving practices theme, 30.3%, 25.4%, 27.0% and 25.2% of smokers reported an earlier intention to quit smoking, respectively. In countries with a variety of HWL designs, such as Canada, the content of warning labels is often varied to broadly educate smokers and nonsmokers about the health consequences of smoking [33, 55]. It is interesting that in this study, labels that had content about cultural practices (such as gifting cigarettes) and labels that included content to support smoke-free spaces were also effective at increasing reported interest in cessation or interest in quitting cigarette use sooner, suggesting that a variety of messages may support the important goal of encouraging quitting. Chinese culture emphasizes the value of collectivism compared with Western cultures [36, 38]. Previous studies indicate that HWLs depicting the harms of SHS on children or women raised Chinese smokers’ intention to quit smoking [25, 37]. Our study also found that smokers are more likely to have an interest in quitting sooner if the HWLs made them more concerned about harming children with SHS after controlling for age, gender, income and educational attainment. Chinese smokers may have a motivation to quit smoking sooner because these HWLs may teach them new health information and make them more concerned about the health consequences of smoking on their family and children. However, smokers in this study reported less interest in quitting sooner if the HWLs were more effective in stopping people from gifting cigarettes. Because gifting cigarettes is a common and complex social practice in China, future studies may collect data regarding smokers’ perception of gifting cigarettes and receiving cigarettes, and the effect of these behaviors on the time to quit. This study has several limitations. First, the research was conducted in three highly developed cities that do not fully represent the geographic and economic development diversity in China. The findings therefore may not be generalizable to the Chinese population living in underdeveloped rural areas or smaller cities. Second, the follow-up assessment in this study was immediate and it is unknown how long the observed effect might last. People may respond differently if they have repeated exposure to HWLs on actual cigarette packages. In addition, intention to quit smoking was self-reported which may not reflect or lead to actual cessation seeking behavior and may be subject to social desirability response bias. Longitudinal studies are needed to further examine the impact of pictorial HWLs on smoking cessation behaviors if pictorial HWLs are implemented in China in the future. The HWLs in this study were developed to be realistic and similar in design to pictorial HWLs used in other jurisdictions. The HWLs studied were examined for specific thematic content. The labels have multiple design features and it is not possible to ascertain what or how each unique feature contributed to the reported results. Thus, findings should be taken considering the HWLs in their entirety. While the data were collected in 2017, the policy context has not changed in these cities in the past 5 years and thus we expect the relationships to hold. Despite these limitations, this study provides important evidence about the impact of pictorial HWLs on smokers’ intention to quit smoking. These findings can inform the Chinese government and other stakeholders about the effectiveness of pictorial HWLs in encouraging smoking cessation.

## Conclusions

This study provides evidence that pictorial HWLs are associated with increased reported intention to quit smoking and the perceived effectiveness of pictorial HWLs was positively associated with smokers’ intention to quit among Chinese smokers in Beijing, Shanghai, and Shenzhen. All themes tested in this study including self-harm from using cigarettes, harming family or children with secondhand smoke, compliance with existing smoke-free policies, and cigarette gift giving practices were associated with increased reported intention to quit, with theme of self-harm having the greatest increase in increased intention. These findings provide support for the Chinese government to design, develop and evaluate a series of pictorial HWLs that can address China-specific culture and tobacco control challenges.

## Data Availability

Data are available upon reasonable request. Requests to access the datasets should be directed to QN, qnian1@jhu.edu.
